# Correlating reductive vanadium oxide transformations with electrochemical N_2_ activation and ammonia formation†

**DOI:** 10.1039/d5cp00554j

**Published:** 2025-06-02

**Authors:** Kabirat Balogun, Qasim Adesope, Stella Amagbor, Agbara Tochi, Adam Vass, Guido Mul, Christoph Baeumer, Georgios Katsoukis, Jeffry A. Kelber

**Affiliations:** a Dept. of Chemistry, University of North Texas Denton TX 76203 USA g.katsoukis@utwente.nl; b MESA+ Institute for Nanotechnology, Faculty of Science and Technology, University of Twente Drienerlolaan 5 7522 NB Enschede The Netherlands

## Abstract

The electrochemical reduction of nitrogen to ammonia (E-NRR) could become an environmentally friendly approach, yet its molecular-scale reaction mechanisms remain difficult to elucidate. Here, we use *in situ* electrochemical infrared reflection–absorption spectroscopy (EC-IRRAS) to examine vanadium oxide electrodes in neutral aqueous electrolyte (pH 7). *Ex situ* XPS reveals that the vanadium oxide electrode initially consists predominantly of V^5+^ species in the form of V_2_O_5_. However, in neutral aqueous electrolyte (pH 7), the surface evolves into anionic *ortho*-, *meta*-, and polyvanadate species at potentials above +0.6 V *vs.* RHE. Upon cathodic sweeping these anionic vanadates undergo progressive reduction toward V_2_O_4_. In an N_2_ saturated electrolyte, subsequent reduction and redeposition of these anionic vanadates remove a distinct vanadyl (V^4+^=O) feature – likely associated with an undercoordinated site, *i.e.* oxygen vacancies or grain boundaries – while the appearance of a broad, red-shifted band suggests the formation of vanadyl intermediates that interact with N_2_. Crucially, we find that ammonia (or ammonium) formation initiates at −0.28 V *versus* RHE, coinciding with a phase transition from V_2_O_4_ to V_2_O_3_ and continues until this transition completes. This onset is accompanied by the appearance of adsorbed N_2_ at −0.28 to −0.38 V *versus* RHE, indicating an associative mechanism. Overall, these findings emphasize the pivotal role of transient redox transitions (V^5+^ → V^4+^ → V^3+^) in enabling N_2_ activation – beyond the static presence of V_2_O_3_ alone – and highlight the promise of vanadium oxides as dynamic platforms for E-NRR.

## Abstract 2

E-NRR on vanadium oxide cathodes in neutral aqueous electrolyte proceeds *via* reductive phase transitions from V^5+^ oxide to V^3+^ oxide forming mixed-valence V^3+^/V^4+^ phases, generating active sites for N_2_ activation and NH_3_ formation.

## Graphical abstract

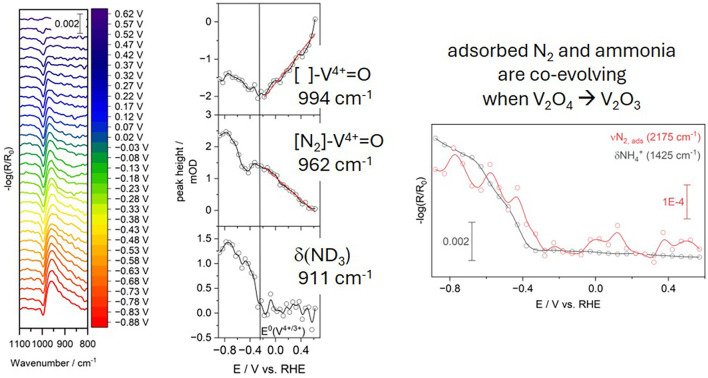

## Introduction

Given the essential role of ammonia in industry and society,^1–4^ the development of sustainable production methods is imperative. Electrochemical nitrogen reduction (E-NRR) presents a sustainable alternative for ammonia production by utilizing renewable energy sources.^1,5,6^ However, the development of highly selective and efficient (*i.e.* low overpotential) electrocatalysts for E-NRR remains a significant challenge. This is primarily due to the competitive hydrogen evolution reaction (HER), which occurs at less negative potentials than E-NRR, typically reducing the selectivity and efficiency of ammonia synthesis.^3,5,7,8^

To address the challenges of HER competition, various transition metal-based materials have been explored for NRR, including oxides, oxynitrides, and carbides of Fe, Cu, V, and Nb.^1,5,8,9^ Both experimental and computational studies on vanadium compounds, including VN,^7,8^ VON,^9,10^ and VO_*x*_,^11,12^ as well as vanadium single-atom catalysts,^13^ have identified the active site as the vanadium center coordinated to lattice oxygen. Additionally, VO_*x*_ has been identified as highly selective for NRR over HER under acidic or neutral reaction conditions.^14^ While numerous computational studies^8,14,15^ propose an associative pathway for nitrogen reduction on metal oxide surfaces – *i.e.*, N_2_ associative adsorption, followed by protonation – experimental validation of this mechanism remains elusive. *In situ* electrochemical Fourier-transform (FT)-infrared spectroscopy has been utilized to investigate the adsorption, dissociation, and hydrogenation of N_2_ on various cathodes.^16–18^ Despite advancements, peak assignments remain ambiguous, and the oxidation state of vanadium cations, along with the roles of surface vanadyl and lattice oxygen during key steps – particularly N_2_ chemisorption and surface nitridation – remains unclear.^19,20^ In this study, we investigated catalyst surface changes and reaction intermediates formed during the E-NRR on nitrogen-free vanadium oxide thin film catalysts using electrochemical FT-infrared reflection–absorption spectroscopy (EC-IRRAS). By identifying reaction intermediates, tracking time-resolved changes in catalyst oxidation states, and detecting new bond formations, we integrated our findings with previous studies to elucidate the NRR mechanism on the vanadium oxide surface. This enabled us to determine the onset potential for vanadium N_2_ interactions, chemisorbed N_2_, and ammonia, correlating these processes to the oxidation state of vanadium.

## Materials and methods

The vanadium oxide thin films used for this study were synthesized at the University of North Texas (UNT) using a method described elsewhere.^12,21^ Briefly, films were deposited using a DC magnetron sputter deposition method in an ultrahigh vacuum chamber with a base pressure of 10^−8^ Torr. The chamber is also equipped with capacity for inductively coupled plasma treatment, and Auger electron spectroscopy (AES) for *in situ* sample analysis.^22^ Films were produced by generating plasma in 4 mTorr Ar gas at 20 W for 20 min at room temperature using a commercially available vanadium metal target (purity 99.7%, Plasma Materials Inc.) mounted on a Meivac/Ferrotac commercially available DC magnetron sputter gun. Samples were analyzed *in situ* using AES. The AES instrument is a commercial single pass cylindrical mirror analyzer with co-axial electron gun operated at 3 keV (Staib, Inc.).

Samples were deposited on glassy carbon substrates for EC-IRRAS experiments. *Ex situ* XPS was done at University of Twente before electrochemical tests (Omicron XM 1000 Al-Kα, monochromatic X-ray source, 1486.6 eV, Omicron EA 125 energy analyzer). The photoemission analyzer angle with respect to the sample normal was 0°. XPS spectra were analyzed using CasaXPS and a Shirley background was used for peak fitting.^23^ XPS spectra were referenced to the O 1s binding energy of lattice oxygen of vanadium oxide at 530.0 eV as popularly reported in literature.^24–26^ In analysis of the vanadium 2p spectra, multiplet splitting due to the final states effects of V photoelectrons was taken into consideration.^25,27^ The complexity associated with the multiplet splitting was resolved by analysing the V 2p peak as previously reported by Coulston *et al.*, 1996.^25,27^ The average oxidation state distribution of V sites can be estimated by applying the equationAverage VOx = 13.82 − 0.68 [O 1s–V 2p_3/2_]where O 1s and V 2p_3/2_ are the binding energy positions of the respective peaks in eV.^27^

For *in situ* electrochemical studies, a Bruker V80v FTIR instrument was used to acquire spectral data. The instrument is made up of an IR source, an MIR polarizer (KRS-5) in an automatic rotational unit capable of p-polarization and s-polarization, a ZnSe IR transparent hemispherical crystal, an A530/V reflection unit for the electrochemical cells, and a medium band MCT detector. The detector is of medium-bandwidth (12 000–600 cm^−1^) cooled with liquid nitrogen during use. The polarizer was set to p-polarization, while the reflective gold mirrors were set at an incident angle of 30°, resulting in a 70–80° refraction angle at the electrolyte/ZnSe interface. These angle settings increased the surface electric field through a “grazing angle” geometry,^28^ thus ensuring amplification of vibrational modes of constituents in the electrolyte near the metal electrode surface. An estimation of the percentage contributions of the vibrational mode intensity *vs.* distance to the electrode can be found in our previous work.^29^ Please note that according to the Fresnel equations *ca.* 96% of the IR beam is reflected at the VOx interface,^30^ meaning that up to 4% of the IR signal may also contain VOx bulk information because of an IR beam penetration depth in the 5 to 6 micrometer range (that may reflect at another VOx interface and, hence, reach the detector). The instrument aperture and resolution were set at 1.5 mm and 8 cm^−1^ respectively. Electrochemical conditions were applied using a VersaSTAT3 potentiostat connected by BNC through a Bruker E525/Z connector box to allow automatic trigger of the potentiostat from the OPUS 3D software to synchronize IR spectra and *I*–*V* data collection. Prior to measurement, the ZnSe crystal was polished, rinsed in an ultrasonic bath, and dried. The working electrode was mounted on a holder with electrical back contact to a copper rod placed in otto configuration (upside-down) in close contact with the crystal in the electrochemical cell compartment filled with 4 ml electrolyte solution. The reference (Ag/AgCl) and counter (Pt wire) electrodes were also inserted to form a 3-electrode system. The working electrode was adjusted until its alignment with the ZnSe crytal produced an amplitude of around 2500. This set-up produced a thin layer of *ca.* 1 to 2 micrometer electrolyte between the working electrode and the ZnSe crystal. Gas was continuously delivered and bubbled through a plastic delivery tube directly into the electrolyte. Scheme S1 (ESI†) shows the schematic representation of the electrochemical FTIR set-up.

Cyclic voltammograms between +1.0 V and −0.3 V *vs.* RHE were run for 50 cycles as a surface cleaning step to remove adsorbed contaminants and generate a clean and stable catalyst surface before electrolyte saturation with gas. Gas was delivered for 30 minutes to ensure saturation before running CV from +0.68 V to −0.83 V. Experiments were carried out in N_2_ saturated, Ar saturated and deuterated electrolyte solution. Obtained spectra were processed using OriginPro. Electrolyte used was 0.1 M NaCl aqueous and deuterated solution. We previously reported the E-NRR activity of vanadium oxide thin films in a 0.1 M Na_2_SO_4_ solution at pH 7.^12^ In the present study, however, we employed a 0.1 M NaCl solution instead. This change was made due to the strongly absorbing asymmetric stretch of the sulfate anion in the region of interest between 1300 and 1000 cm^−1^.

## Results

### Thin film deposition and characterization.

**Fig. 1:**
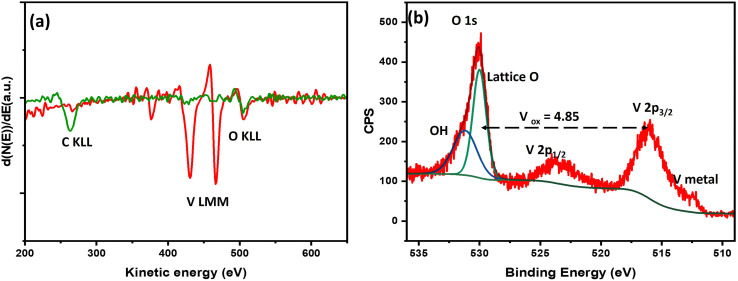
(a) *In situ* AES spectra of a vanadium thin film on glassy carbon electrode (green trace before, red trace after deposition). (b) *Ex situ* XPS spectra of a vanadium thin film on glassy carbon prior to EC-IRRAS measurement, exhibiting metallic and oxidized vanadium species.

*In situ* AES spectra of a vanadium film on glassy carbon substrate before and after deposition are shown in Fig. 1a. The spectrum before deposition, in green, shows a C KLL peak at 265 eV and an O KLL feature at 503 eV that can be assigned to C and adsorbed oxygen from the glassy carbon substrate prior to V deposition.^23^ The spectrum in red was acquired after vanadium deposition, and shows V LMM and O KLL peaks only, without a C KLL peak. The presence of a O KLL peak with such low intensity can be explained to be due to VO_*x*_ formed due to residual oxygen in the deposition chamber at a base pressure of ∼10^−8^ Torr. The thickness of the film is estimated to be 50 nm.

*Ex situ* XPS analysis prior to electrochemical testing and IRRAS measurements is shown in Fig. 1b. The average V oxidation state was estimated to be 4.85, suggesting highly oxidized vanadium oxide at the surface with the presence of some oxygen vacancies. From the spectral features, the V 2p_3/2_ peak maximum is centered above 516 eV which is a well-reported binding energy for V in +5 oxidation state.^24,26,27^ The peak at 512.3 eV corresponds to V(0), *i.e.* V metal, thus suggesting a thin layer of vanadium oxide on pure V metal. Thus, the sample exhibits a layered VO_*x*_/V structure.^26^ The long ambient exposure of the sample during shipping led to such oxidation at the surface.

### *In situ* EC-IRRAS: staircase cyclic voltammetry (SCCV)

Staircase cyclic voltammetry (SCCV) was performed in N_2_- and Ar-saturated 0.1 M NaCl aqueous solutions (Fig. S1, ESI†) with a 37.25 s hold time per 100 mV step, corresponding to an effective scan rate of 1.34 mV s^−1^. FTIR scans were collected *in situ*. An initial cathodic current, detected in both N_2_ and Ar at +0.5 V *versus* RHE, corresponds to the reversible reduction of vanadium oxide. A second irreversible reduction current is observed predominantly in N_2_-saturated electrolyte from −0.1 V *versus* RHE. Due to the open configuration of our setup, rapid exchange between ambient air and Ar occurs, preventing the complete removal of N_2_ from the electrolyte (see Note S1 in the ESI†). Hence, the experiments denoted with Ar and N_2_ therefore reflect experiments with ‘low’ and ‘high’ concentrations of N_2_.

Prior to further interpretation, we performed a detailed analysis of the infrared spectra to rule out contributions from potential contaminants (*e.g.*, NO, N_2_O, and NO_2_). Further details are provided in Note S2 in the ESI.†

#### 1100 to 800 cm^−1^: V–O and V–N vibrations

**Fig. 2:**
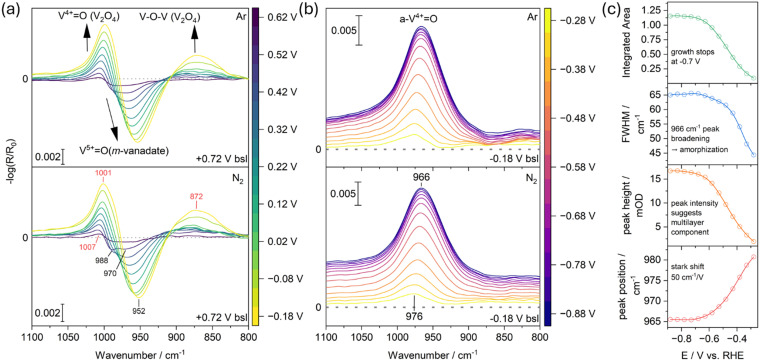
EC-IRRAS data illustrating the reduction of vanadium oxide under cathodic polarization in D_2_O (0.1 M NaCl). (a) Spectra recorded in Ar-purged (top) and N_2_-purged (bottom) solutions using +0.72 V *vs.* RHE as the baseline. (b) Spectra referenced to −0.18 V *vs.* RHE, highlighting the evolution of key vibrational features. (c) Gaussian fitting parameters for the spectra in the bottom panel of (b). Insets summarize the main conclusion. “m-vanadate” denotes the V^5+^=O stretching vibrations of anionic dissolved vanadium oxide species, while “a-V^4+^=O” refers to the vanadyl vibrations that emerge as adjacent V^4+^ centers are reduced to V^3+^, leading to subsequent amorphization.

In Fig. 2, we focus on the IR region between 1100 and 800 cm^−1^ to highlight the V=O stretching vibrations characteristic of vanadate (V^5+^) and vanadyl (V^4+^) species.^31,32^ The spectra were recorded in deuterated water to minimize interference from the H_2_O libration modes typically observed below 1000 cm^−1^. Terminal V=O bonds, located at the edges of the vanadium polyhedra, serve as reliable markers of the oxidation state.^33,34^ Our working electrode was conditioned at +0.72 V *versus* RHE (+0.31 V *versus* SHE), which served as the experimental baseline. At this potential, the vanadium oxide electrode predominantly undergoes corrosion, resulting in the formation of anionic species such as HVO_4_^−^ (orthovanadate) and HV_10_O_28_^5−^ (polyvanadate), in agreement with the Pourbaix diagram for the V–H_2_O system at pH 7.^35^

Upon cathodic sweeping, in Fig. 2a a broad negative peak appears at 988 cm^−1^ at +0.62 V *versus* RHE, gradually shifting to 952 cm^−1^ as the potential is swept to −0.23 V *versus* RHE. We attribute these features to the redeposition of dissolved vanadate species that exhibit V^5+^=O vibrations significantly red-shifted compared to solid V_2_O_5_:^36^ the initial peak is associated with polyvanadate, and the shift to 952 cm^−1^ corresponds to the redeposition and reduction of meta- and orthovanadate. Given the initial IR spectra baseline at +0.72 V already includes features from anionic vanadate species at the interface, their reduction would result in reduced IR peak intensity resulting in a negative peak in the IR spectra as observed in Fig. 2a. Importantly, no negative peaks are observed at 1016 cm^−1^ (TO mode) or 1035 cm^−1^ (LO mode), which would indicate the reduction of solid V_2_O_5_.^37^ Concurrent with these changes, new broad, kinetically co-evolving bands emerge with maxima at 872 cm^−1^ and 1001 cm^−1^. We assign these bands to the formation of V^4+^–O and V^4+^=O stretching vibrations of V_2_O_4_, respectively.^34,38^

At −0.18 V *versus* RHE, a marked change in spectral evolution is observed: the reduction of vanadate species reflected by the growth of the V^4+^=O feature at 1001 cm^−1^ stops. In Fig. 2b, spectra are referenced to a baseline at −0.18 V *versus* RHE, which clearly reveals the emergence of a dominant band at 966 cm^−1^. We assign this feature to the formation of oxovanadium species (V^3+^–O), consistent with the phase transitions expected from the Pourbaix diagram.^33^ Since V_2_O_3_ is not known to exhibit V=O double bonds, we cannot exclude that the 966 cm^−1^ band instead represents a red-shifted V^4+^=O vibration influenced by neighboring V^3+^ sites, where the shift reflects bond weakening.^39^ The continued presence of the 1001 cm^−1^ band suggests that the V^4+^=O species remain intact, while the new oxovanadium species form at distinct surface or subsurface sites.

To elucidate the structural changes occurring in the vanadium oxide electrode from −0.18 V *versus* RHE onward, we fitted the 966 cm^−1^ peak using a Gaussian function (see Fig. 2c). First, the peak maximum shifts from 976 to 966 cm^−1^, corresponding to a stark shift of approximately 50 cm^−1^ V^−1^, confirming the bond weakening of the V^4+^=O species when the electron density is increasing. Second, the peak height increases to 17 mOD, indicating the formation of V=O bonds not only at the surface but also within the subsurface region. This suggests that structural changes, such as subsurface exposure or increased surface roughness, generate additional V=O modes. For comparison, previous studies on ultrathin alumina electrocatalyst overlayers reported a linear LO mode intensity–thickness dependence of 3 mOD nm^−1^,^40^ implying that a monolayer alone would not account for the observed intensity. Third, the full width at half maximum (FWHM) steadily increases from about 40 to 65 cm^−1^, suggesting progressive amorphization or increase in grain boundaries.^34^ Finally, the integrated peak area stabilizes at −0.7 V, indicating that the overall process reaches completion at that potential. Consequently, we attribute the observed spectral features to the formation of VO_*x*_ polyhedra, denoted as a-V^4+^=O, that are defect- and grain-boundary-rich.^34,41^

**Fig. 3:**
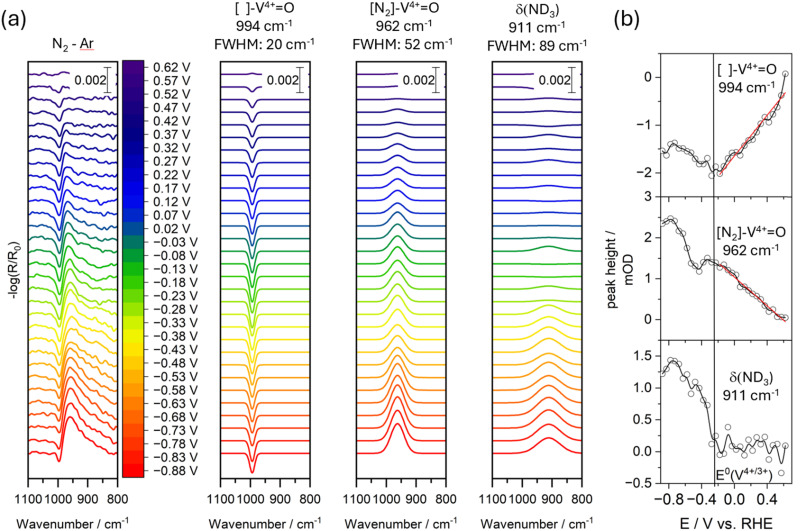
(a) EC-IRRAS difference spectra generated by subtracting the spectra recorded under Ar purging (top panels in Fig. 2) from those obtained under N_2_ purging (bottom panels of Fig. 2). The Gaussian fits of the 994 cm^−1^, 962 cm^−1^, and 911 cm^−1^ bands as a function of applied potential are shown next to it. Peak assignments and FWHM are shown above. (b) Peak maxima *versus* applied potential: the red line shows the linear anti-correlation of the bands at 994 cm^−1^ and 962 cm^−1^. The black vertical line indicates the V^4+^/V^3+^ redox potential under the experimental conditions. Experiments have been done in D_2_O (0.1 M NaCl).

To ensure that subtle differences between the Argon-purged and N_2_-purged experiments were captured, we subtracted the spectra from the Argon-purged experiment from those obtained under N_2_ purging as shown in Fig. 3a. The spectral evolution over time and potential is highly dependent on the electrode pretreatment and initial conditions. These factors were carefully controlled and are taken into account in our data processing to ensure that the subtraction accurately reflects the impact of dissolved N_2_.

In the resulting difference spectra, a subtle, sharp minimum with a full width at half maximum (FWHM) of 20 cm^−1^ becomes apparent at 994 cm^−1^, emerging with an onset at +0.52 V *versus* RHE. Concurrently, a broad feature appears at 962 cm^−1^ with a FWHM of 52 cm^−1^. These observations suggest that N_2_ interacts with the vanadium oxide electrode during the redeposition of corroded vanadate species. We tentatively interpret the sharp minimum as a removal of a specific undercoordinated [ ]-V^4+^=O vanadyl moiety associated with a nearby oxygen vacancy or a reactive grain boundary. The broad positive band is consistent with a red shift of this vanadyl feature. This shift could reflect the filling of the vacancy with a nitrogen atom, leading to the formation of [N]–V^4+^=O or interaction of the vacancy with N_2_ ([N_2_]–V^4+^=O). To provide a more direct evidence for these processes, further experiments conducted at higher N_2_ concentrations (*e.g.*, elevated pressures), with enhanced time resolution and varied time-potential profiles, will be essential.

Another broad feature emerges at 911 cm^−1^ with a FWHM of 89 cm^−1^, which we attribute to the NH_2_ bending mode of neutral (unprotonated) ammonia. We discuss this feature in greater detail in the next section.

Experiments performed in normal water (see Fig. S2, ESI†) are significantly distorted by dynamic changes in the water vibration modes below 1000 cm^−1^. Although these experiments are not directly comparable to those in deuterated water – since the corrosion and redeposition processes between +0.6 V and −0.2 V *vs.* RHE are not well captured – we nonetheless observe a weak band emerging at 980 cm^−1^. We attribute this band to the interaction between a vanadyl moiety and N_2_ as previously mentioned.

To obtain more robust kinetic information, we deconvoluted the observed features using three Gaussian functions – keeping the frequency positions and FWHM fixed – and extracted the peak maxima, as shown in Fig. 3b. The negative growth of the band at 994 cm^−1^ proceeds until −0.28 V *versus* RHE, which coincides with the V^4+^/V^3+^ redox potential and marks the onset of ammonia formation. Interestingly, the band at 962 cm^−1^ initially follows similar growth kinetics (see the red line in Fig. 3b); however, at −0.28 V *vs.* RHE there is a transient decrease in its intensity as ammonia production starts, followed by a subsequent increase. This behavior suggests that the feature at 962 cm^−1^ is correlated with ammonia formation and reflects the interaction of chemisorbed N_2_ or nitride species. At −0.7 V, ammonia formation comes to a halt, which matches the a-V^4+^=O growth stop.

These observations indicate that the formation of [N_2_]– or [N]–V=O is not driven solely by the applied potential; instead, the redeposition and reduction of vanadate (V^5+^) to vanadyl (V^4+^) and V^3+^ appear to be predominant factors. The onset of ammonia formation occurs at a potential nearly identical to that for the V_2_O_4_-to-V_2_O_3_ phase transition, and ceases once this transition is complete. This close correlation implies that the reductive transformation may play a critical role in facilitating N_2_ reduction. Consequently, future studies should focus on (1) investigating the energetics and oxygen vacancy generation associated with the corrosion and redeposition of vanadate species, and (2) elucidating the influence of the V_2_O_4_-to-V_2_O_3_ phase transition on ammonia formation. These efforts will be essential for understanding the mechanistic pathways of N_2_-to-NH_3_ conversion in our system.

#### 1700 to 1100 cm^−1^: formation of ammonium/ammonia

**Fig. 4:**
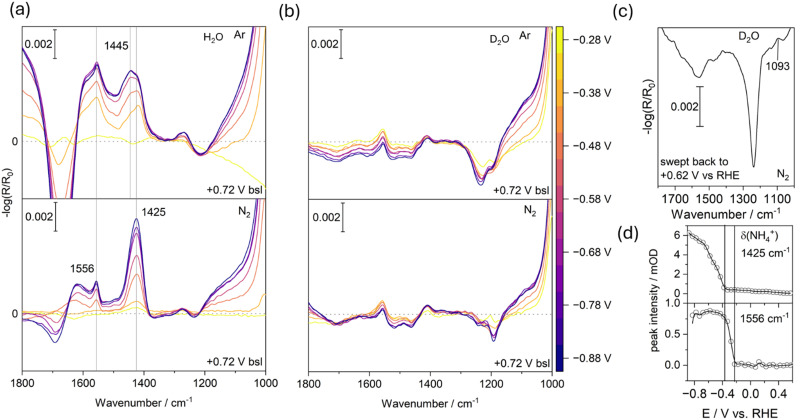
(a) EC-IRRAS spectra showing the formation of ammonium, identified by the NH_2_ bending mode at 1425 cm^−1^, during cathodic polarization in H_2_O (0.1 M NaCl) using +0.72 V *vs.* RHE as the baseline. The top panel shows measurements under Ar, while the bottom panel shows data collected under N_2_. (b) Control experiment performed in D_2_O reveals no NH_2_ nor ND_2_ bending modes of ammonium, supporting that ammonia formation occurred earlier as evidenced in Fig. 3. (c) Anodic back sweep following the D_2_O experiment in (b), showing the emergence of ND_4_^+^ attributed to a shift in the interfacial pD. (d) Potential- and time-resolved evolution of the ammonium feature alongside a band at 1556 cm^−1^, tentatively assigned to hydroxylamine formation.

Fig. 4a displays the fingerprint region (1800–1000 cm^−1^) of the SCCV experiments, from −0.28 V to −0.88 V *versus* RHE in normal water. A new band appears at 1425 cm^−1^ under both Ar- and N_2_-purged conditions – although its intensity is markedly higher in the N_2_-purged experiment (see Note S1, ESI†). We attribute this feature to the formation of ammonium (NH_4_^+^), a conclusion further supported by the reference ATR-FTIR spectrum of ammonium m-vanadate dissolved in water presented in Fig. S3 (ESI†). Additionally, bands at 1556 cm^−1^ and 1273 cm^−1^ appear at −0.3 V *versus* RHE and do not further grow beyond that potential. These are tentatively assigned to the NH_2_ bending and wagging modes of hydroxylamine, respectively.^42^ The small shoulder at 1445 cm^−1^ appears exclusively in the Ar-purged experiment, and we do not yet have a definitive explanation for its origin.

Fig. 4b presents the same experiment performed in deuterated water. As expected, the ammonium band normally observed at 1425 cm^−1^ is absent. However, we also do not detect the anticipated ND_4_^+^ feature around 1090–1070 cm^−1^ (which should be shifted by a factor of approximately 1.31).^43,44^ Instead, as discussed earlier, we observe the formation of ammonia-d_3_, which typically exhibits a symmetric bending mode in the 870–840 cm^−1^ range (the more intense asymmetric bending mode is usually superimposed by water bending modes).^45^ In our case it is blueshifted to 911 cm^−1^, which commonly occurs when ammonia acts as a ligand and, hence, may be interacting with vanadium cations.^46^ The NH bending mode of cationic ammonium is much more intense than that of neutral ammonia. Although our experimental setup does not allow precise control of the interfacial pH, an anodic sweep should reacidify the interface, thereby promoting the conversion of ammonia to ammonium. This effect is demonstrated in Fig. 4c, where a new band emerges at 1093 cm^−1^ upon cycling back to the initial potential at the start of the SCCV, which we attribute to the ND bending mode of ammonium-d_4_.^43^

Fig. 4d shows the spectral evolution of the bands at 1425 cm^−1^ and 1556 cm^−1^, respectively. The formation of ammonium coinsides with the formation of ammonia in the deuterated water experiment (Fig. 3). The band at 1556 cm^−1^ has an earlier onset at −0.2 V, does not exhibit any growth and therefore might also originate from a carboxylate impurity.

#### 2400 to 1800 cm^−1^: chemisorbed N_2_

**Fig. 5:**
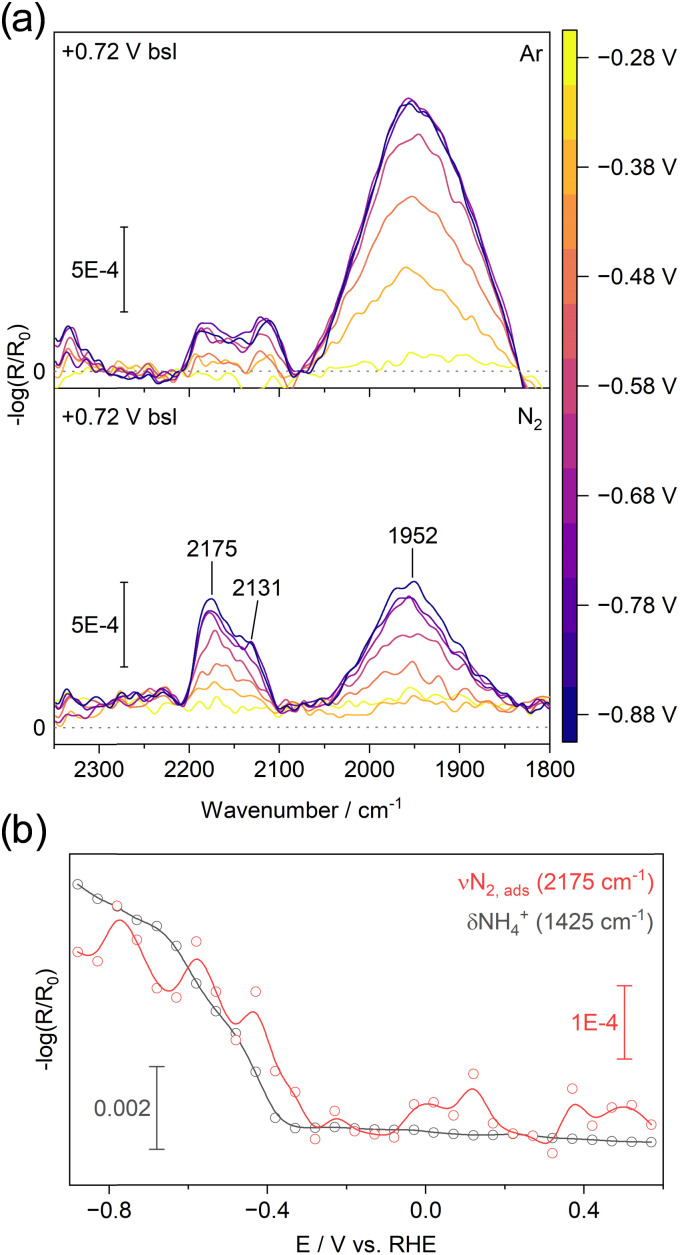
(a) EC-IRRAS spectra recorded during cathodic polarization in H_2_O (0.1 M NaCl), using +0.72 V *vs.* RHE as the baseline. The top panel shows measurements under Ar, while the bottom panel shows data collected under N_2_. The band at 1952 cm^−1^ is attributed to the first overtone of vanadyl (V=O) vibrations. The bands at 2175 and 2131 cm^−1^ are assigned to end-on adsorption of N_2_ on the vanadium oxide surface. (b) Potential trace of the intensities at 2175 cm^−1^ and 1425 cm^−1^ (from Fig. 4d).

In the presence of N_2_, new vibrational bands emerge in both normal (Fig. 5) and deuterated water (Fig. S4, ESI†) at −0.43 V *versus* RHE. A broad band centered at 1952 cm^−1^ is observed, which we assign to the first overtone of the amorphous V^4+^=O stretching vibration appearing at 976 cm^−1^.^47^ In addition, two distinct peaks at 2175 cm^−1^ and 2131 cm^−1^ are detected. Based on IRRAS studies of N_2_ adsorption on Li-preadsorbed Ni{110},^48^ theoretical calculations for N_2_ chemisorption on V(110),^49^ and DRIFTS studies on aluminovanadate oxynitride catalysts^46^ these bands are assigned to the N=N stretching mode of chemisorbed N_2_ on vanadium sites, rendered IR-active by symmetry breaking. Because IRRAS selectively detects vibrations perpendicular to the surface, these results suggest that adsorbed N_2_ adopts either a head-on or tilted orientation relative to the vanadium oxide surface. This is consistent with previous near ambient pressure XPS studies^21^ of V oxide surfaces in the presence N_2_ and H_2_O vapor, and with previous DFT calculations^14^ indicating that V^3+^ surface sites most strongly interact with N_2_.

Although we cannot completely exclude the formation of nitrile species – which can display similar doublet-like features in this spectral region^50,51^ – it would require the prior cleavage of N_2_ followed by a reaction with carbon impurities.^52^ We also cannot exclude azides that may evolve due to the reaction of N_2_ with nitride.^46^ Nevertheless, all cases involve N_2_ chemisorption as a key step. The observed spectral features emerge at around −0.38 V *versus* RHE slightly after the transition from V^4+^ (V_2_O_4_-type) to V^3+^ (V_2_O_3_-type) starts and concurrent with ammonium formation (Fig. 5b). The appearance of adsorbed N_2_ when V^3+^ centers are present has been observed previously.^10,12^

The top panel in Fig. 5 presents a similar experiment conducted in an Ar-saturated electrolyte, which confirms the N_2_ concentration dependence of the features at 2175 cm^−1^ and 2131 cm^−1^. As noted earlier, due to the open configuration of our setup, rapid exchange between ambient air and Ar occurs, preventing the complete removal of N_2_ from the electrolyte (see Note S1, ESI†).

## Discussion

The observed spectral changes reveal a complex evolution of vanadium oxide during electrochemical reduction. Under our experimental conditions at pH 7, the electrode corrodes and gradually dissolves to form anionic species – including m-vanadate, orthovanadate, and polyvanadate – at potentials more positive than +0.6 V *versus* RHE at the interface. Upon reduction and redeposition of these anionic species, we can observe that N_2_ interactions are leading to a redshift of a vanadyl feature. Notably, when the phase transition between V_2_O_4_ and V_2_O_3_ occurs at −0.28 V *versus* RHE, ammonia/ammonium formation begins and continues until the phase transition is finalized. Concurrently, this phase transition is accompanied by the formation of adsorbed N_2_.

These transformations in the vanadium oxide are critical for N_2_ reduction. As V^5+^ is reduced to V^4+^ and then to V^3+^, the lattice undergoes structural reorganization, resulting in progressive amorphization of the surface and the creation of undercoordinated vanadium sites with additional oxygen vacancies and grain boundaries. These changes eventually facilitate the adsorption and dissociation of N_2_ in an associative mechanism. The process is inherently disordered and appears to extend into the subsurface of the electrode, as evidenced by the emergence of new vanadyl features even as reduction advances toward V^3+^.

The weak spectral features suggest that the current experimental conditions do not adequately capture the reaction kinetics. In particular we cannot observe the protonation and dissociation of N_2_, which must be on much shorter time-scales. To obtain more robust kinetic data, it is necessary to operate under higher pressure conditions, enhance time resolution, and incorporate chronoamperometric measurements as well as pulsed electrolysis experiments. The correlation that exists between the phase transition and the conversion of N_2_ to ammonia appears to be transient, requiring both a rapid, abundant supply of N_2_ at the electrode and potentially continuous phase switching.

## Conclusions

In this study, we investigated the electrochemical nitrogen reduction reaction (E-NRR) on vanadium oxide electrodes under ambient conditions using *in situ* EC-IRRAS. Our results reveal that at pH 7 the electrode corrodes and gradually dissolves to form anionic species – including m-vanadate, orthovanadate, and polyvanadate – at potentials more positive than +0.6 V *vs.* RHE. We observe that the presence of N_2_ upon reduction and redeposition of these dissolved species, leads to the removal of a distinct vanadyl mode that may originate from oxygen vacancies and grain boundaries. The appearance of a broad redshifted band suggests that N_2_ interacts with this ‘defect’. When the phase transition between V_2_O_4_ and V_2_O_3_ occurs at −0.28 V *versus* RHE, ammonia/ammonium formation begins and continues until the phase transition is finalized. Concurrently, this phase transition is accompanied by the formation of adsorbed N_2_.

Ammonia/ammonium formation initiates at −0.28 V *versus* RHE, coinciding with the phase transition from V_2_O_4_ to V_2_O_3_, and continues until the transition is complete. This observation, along with the formation of adsorbed N_2_, implies an associative reduction mechanism, albeit we do not observe the intermediate protonation and dissociation steps.

Overall, our findings indicate that dynamic redox transitions from V^5+^ through V^4+^ to V^3+^ are critical for N_2_ activation. This suggests that the kinetics of ammonia formation are transiently dependent on these phase transitions rather than solely on the presence of a distinct V_2_O_3_ phase. In summary, this work underscores the significant influence of redox transitions and applied potential on the mechanistic pathways of E-NRR, highlighting the potential of vanadium oxides as platforms for nitrogen reduction and the importance of *in situ* techniques for elucidating reaction dynamics and catalyst behavior under operational conditions.

## Author contributions

Kabirat Balogun: investigation, methodology, data curation, formal analysis, writing (original draft, review and editing). Qasim Adesope: investigation, methodology. Stella Amagbor: methodology, investigation, writing (review and editing). Agbara Tochi: methodology, investigation. Adam Vass: investigation, methodology, data curation. Guido Mul: conceptualization, and resources. Christoph Baeumer: resources, writing (review and editing). Georgios Katsoukis: investigation, methodology, data curation, formal analysis, writing (original draft, review and editing), conceptualization, validation, resources, supervision. Jeffry A. Kelber: writing (original draft, review and editing), conceptualization, resources, supervision.

## Conflicts of interest

There are no conflicts to declare.

## Supplementary Material

CP-027-D5CP00554J-s001

## Data Availability

The raw and processed data supporting this study will be publicly available on Zenodo upon acceptance of the manuscript, with a DOI assigned at that time. All data presented in the figures are derived from this dataset.
