# Supplementary material for: Correlating reductive vanadium oxide transformations with electrochemical N2 activation and ammonia formation
Source: Phys Chem Chem Phys. 2025 Jun 2;27(26):13836–44. doi: 10.1039/d5cp00554j (PMC12150205; doi:10.1039/d5cp00554j)
Supplement: CP-027-D5CP00554J-s001 [file CP-027-D5CP00554J-s001.pdf]

This journal is © the Owner Societies 2025

Supporting Information for

Correlating Reductive Vanadium Oxide Transformations with Electrochemical N_2 Activation and Ammonia Formation

Kabirat Balogun[1‡], Qasim Adesope[1], Stella Amagbor[1], Agbara Tochi[1], Adam Vass[2], Guido Mul[2], Christoph Baeumer[2], Georgios Katsoukis[2,], and Jeffry A. Kelber[1,*]*

[1]Dept. of Chemistry, University of North Texas, Denton, TX 76203

[2]MESA+ Institute for Nanotechnology, Faculty of Science and Technology, University of Twente, Drienerlolaan 5, 7522 NB Enschede, The Netherlands

Supplementary note 1: Estimation of N_2 Ingress via Diffusion in an Open 10 cm-Tall Tube

Figure S1 shows a schematic of the electrochemical FT-IR setup and it can be seen that the electrolyte is exposed to ambient air from the top. In our setup, 4 mL of electrolyte is contained at the bottom of a cylindrical tube (2 cm diameter) and is continuously purged with argon from the bottom at 30 mL/min. The tube has a total height of 10 cm, which is exposed to ambient air at the top. Although argon is heavier than air and tends to fill the tube up to the 10 cm mark, ambient air continuously diffuses downward from the open top, leading to N_2 ingress into the headspace and eventually into the electrolyte.

Geometry and system parameters

Tube diameter = 2 cm → cross-sectional area is ca. 3.14 cm^2

Electrolyte volume = 4ml → height 1.27 cm → headspace is 8.73 cm

Argon molar flow = 0.5 ml/s = $2.08 \cdot 10^{-5}$ mol/s

Diffusive N_2 Influx from the Open Top

Ambient air contains 78% N_2. Thus, the N_2 concentration is 0.0317 mol/cm^3.

We assume the argon-filled headspace initially has negligible N_2, so the concentration difference driving diffusion is DC≈3.17×10^{-5} mol/cm^3. Using Fick's first law, the diffusive flux J is: $J=D_{N2}\cdot(DC/L)=1.27\cdot10^{-5}$ mol/cm^2s ($D_{N2,air}$=0.2 cm^2/s; [1] boundary layer thickness L is assumed 0.5 cm)

Multiplying it with the tube cross-sectional area yields $\dot{n}_{N2,diff}=4\cdot10^{-5}$mol/s. We need to put $\dot{n}_{N2,diff}$ into perspective of $\dot{n}_{Ar,flow}=2.08\cdot10^{-5}$mol/s:

Although Ar is denser than air and tends to fill the tube, the open top allows ambient air to continuously diffuse downward. Our over the envelope calculations indicate that, under these conditions, the steady-state headspace reaches an N_2 mole fraction of about 66%, which results in an equilibrium concentration of dissolved N_2 that is approximately 15% lower than the fully air-saturated level. While the presence of electrodes reduces the effective tube diameter and may further limit the ingress of N_2, it remains evident that even with a vigorous argon purge, continuous diffusive ingress prevents complete N_2 removal. This analysis demonstrates that under our experimental conditions - in a 10 cm-tall tube containing 4 mL of electrolyte - the open configuration precludes complete displacement of N_2 by Ar purging alone, underscoring the critical importance of cell geometry and electrode placement in in-situ electrochemical spectroscopic studies.

Supplementary note 2: Excluding contamination reactions

To further exclude the possibility of contamination from the N_2 gas stream, we considered potential contributions from N_2O (ca. 2245 ± 10 cm^{-1})[2] and NO (ca. 1800 ± 10 cm^{-1} on NiO{100}) [3]. However, these modes are not present in our spectra. On metallic nickel and ruthenium, NO can absorb between 1400–1600 cm^{-1}, and in the presence of oxygen, it can form NO_2, which has an N-O stretch at ca. 1600–1650 cm^{-1}. [4,5] To verify whether such species contribute to our spectra, we repeated the experiment in deuterated water to eliminate interference from the water bending mode. Two weak features at 1550 cm^{-1} and 1410 cm^{-1} appeared in both Ar- and N_2-saturated electrolyte, indicating they do not originate from NO_2 adsorption. Instead, we assign these peaks to the asymmetric and symmetric stretches of carboxylate-containing trace impurities (Figure 4b). We can also rule out vanadium carbonyl formation from trace CO impurities, since they produce vibrational feature below 2100 cm^{-1}.[6]

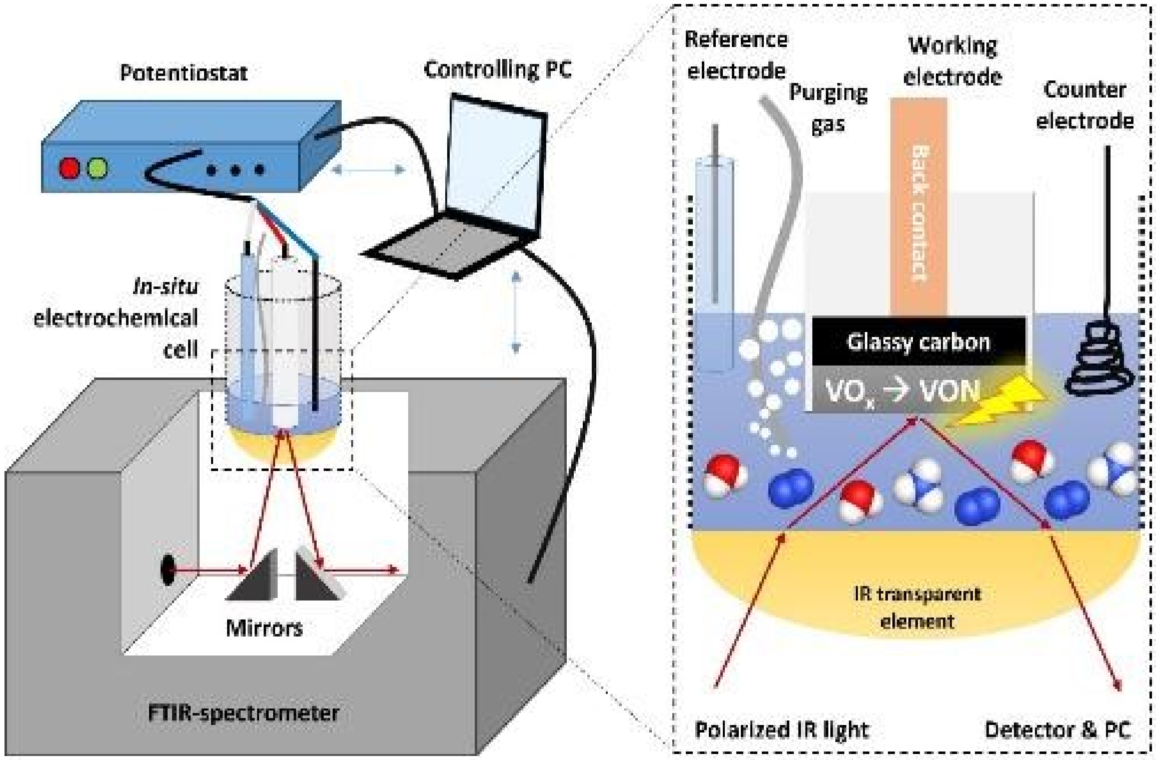

Scheme S1: Schematic for EC-IRRAS instrument set-up

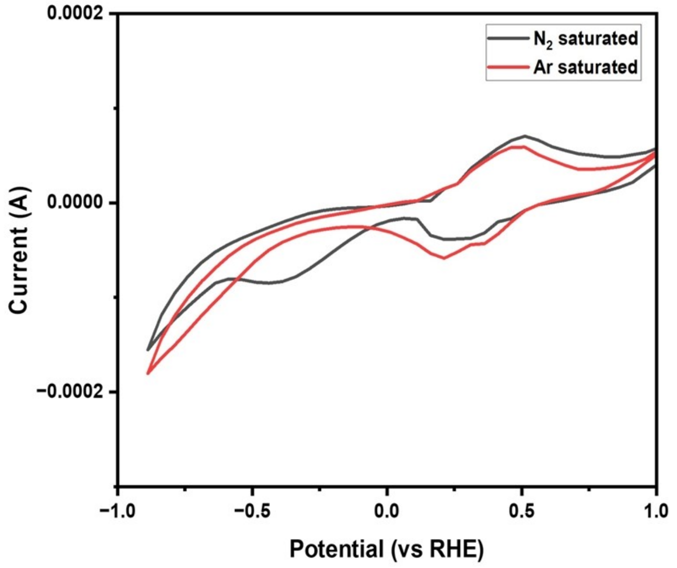

Figure S1: Staircase cyclic voltammogram of the EC-IRRAS measurement of VO_x in N_2-saturated 0.1 M NaCl aqueous electrolyte (black trace) and in Ar (red trace). The hold time was 37.25 s per 100 mV step which leads to an effective scan rate 1.34 mV/s. Small current and potential variations stem from the vanadium oxide placement on the ZnSe crystal, creating a variable 1–2 µm electrolyte film that affects mass transport and causes differences between the applied and interfacial pH-affected potentials.

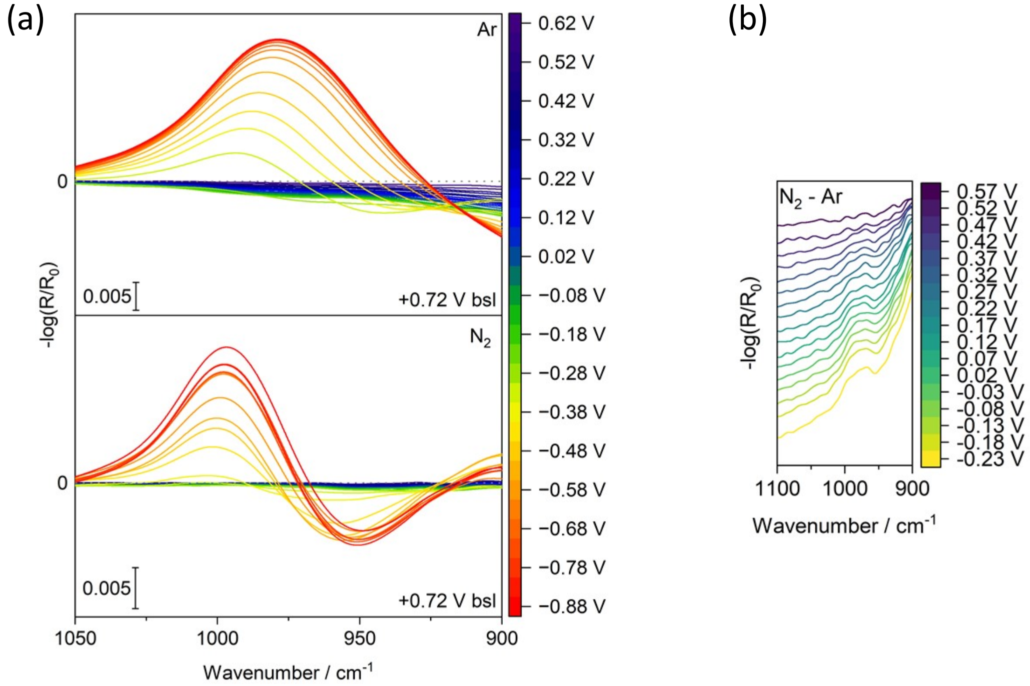

Figure S2: EC-IRRAS data illustrating the reduction of vanadium oxide under cathodic polarization in H_2O (0.1 M NaCl). (a) Spectra recorded in Ar-purged (top) and N_2-purged (bottom) solutions using +0.72 V vs. RHE as the baseline. (b) EC-IRRAS difference spectra generated by subtracting the spectra recorded under Ar purging from those obtained under N_2 purging.

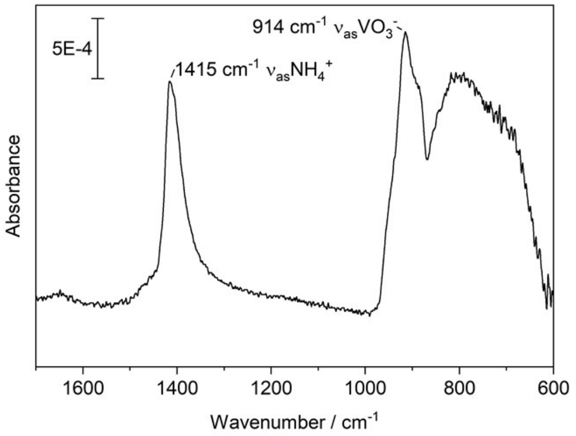

Figure S3: ATR-FTIR of ammonium m vanadate suspended in water.

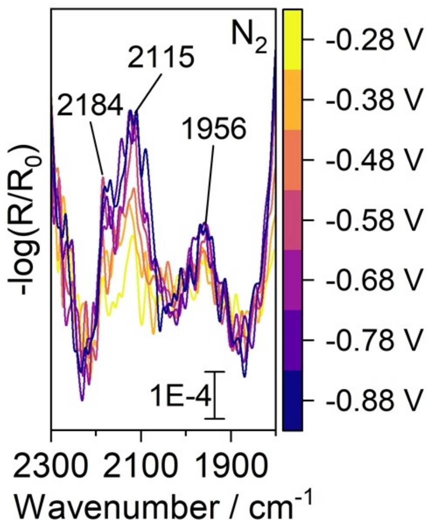

Figure S4: EC-IRRAS spectra recorded during cathodic polarization in D_2O (0.1 M NaCl), using +0.72 V vs. RHE as the baseline in N_2. Baseline correction was performed using an asymptotic least squares method because it was a heavily sloped/wavy spectrum in this region.

References

1. W. J. Massman, *Atmos. Environ.* 1998*,* ***32***, 1111–1127.
2. M. Minissale, G. Fedoseev, E. Congiu, S. Ioppolo, F. Dulieu, and H. Linnartz, *Phys. Chem. Chem. Phys.* 2014*,* ***16***, 8257–8269.

3. H.E Sanders, P. Gardner, D. A. King and M. A. Morris, *Surf. Sci.,* 1994 ***304*(1)**, 159–167.
4. M. Takagi-Kawai, M. Soma, T. Onishi and K. Tamaru, *Can. J. Chem.* 1980, ***58***.
5. W. Erley, *Surf. Sci.,* 1998 ***205*(1)**, L771–L776.
6. B. Immaraporn, N. Magg, S. Kaya, J. Wang, M. Bäumer and H. Freund, *Chem. Phys. Lett. ,* 2004, ***392*(1)**, 127–131.
